# Evaluation of insemination, blood feeding, and *Plasmodium vivax* infection effects on locomotor activity patterns of the malaria vector *Anopheles darlingi* (Diptera: Culicidae)

**DOI:** 10.1007/s00436-023-08053-5

**Published:** 2023-12-07

**Authors:** Alessandra da Silva Bastos, Najara Akira Costa dos Santos, Alice Oliveira Andrade, José Daniel Costa Pontual, Jéssica Evangelista Araújo, Jansen Fernandes Medeiros, Maisa da Silva Araújo

**Affiliations:** 1 Plataforma de Produção E Infecção de Vetores da Malária (PIVEM), Laboratório de Entomologia, Fiocruz Rondônia, Porto Velho, Rondônia Brazil; 2 Programa de Pós-Graduação Em Biologia Experimental, Fundação Universidade Federal de Rondônia, FIOCRUZ Rondônia, Porto Velho, Rondônia Brazil; 3 Programa de Pós-Graduação Em Saúde Pública, Faculdade de Saúde Pública, Universidade Federal de São Paulo, São Paulo, SP 01246-904 Brazil; 4 Programa de Pós-Graduação em Conservação e uso de Recursos Naturais–PPGReN, Fundação Universidade Federal de Rondônia, Porto Velho, Rondônia Brazil; 5 Laboratório de Pesquisa Translacional E Clínica, Centro de Pesquisa Em Medicina Tropical, Porto Velho, Rondônia Brazil

**Keywords:** Locomotor activity, *Anopheles darlingi*, Flybox, Blood feeding, Insemination, *Plasmodium vivax*

## Abstract

Circadian behavioral patterns in mosquitoes can be observed through their locomotor activity, which includes fundamental behaviors such as foraging, mating, and oviposition. These habits, which are fundamental to the life cycle of *Anopheles* mosquitoes, are closely related to pathogen transmission to humans. While rhythmic cycles of locomotor activity have been described in *Anopheles* species, no studies have been conducted on *Anopheles darlingi* species, the main malaria vector in the Amazon region. The aim of this study was to investigate how insemination status, blood meal, and *Plasmodium vivax* infection affect the locomotor activity of *An. darlingi*. The experiments were performed with 3- to 10-day-old *An. darlingi* females, which had been fed with 15% honey solution. These mosquitoes were obtained from the Malaria Vector Production and Infection Platform (PIVEM)/FIOCRUZ–RO. The experimental groups were divided into four categories: virgin vs. inseminated, unfed virgin vs. blood-fed virgin, unfed inseminated vs. blood-fed inseminated, and infected blood vs. uninfected blood. Locomotor activity was monitored using the Flybox equipment, capturing images that were subsequently converted into video to measure the insect activity, using PySoLo software. The periodicity and rhythmicity of mosquito locomotor activity were analyzed using MatLab® software. The locomotor activity of *An. darlingi* females showed a nocturnal and bimodal pattern under LD conditions. When comparing the insemination states and blood meal, there was a reduction in the locomotor activity in inseminated and blood-fed females. However, the *P. vivax*^+^ infection did not increase locomotor activity of *An. darlingi* species.

**Supplementary Information:**

The online version contains supplementary material available at 10.1007/s00436-023-08053-5.

## Background

Locomotor activity of insects, mosquitoes included, follows a circadian rhythm that is regulated by molecular clocks and can be modulated by extrinsic factors, such as light and temperature (Meireles-Filho and Kyriacou 2013). At the beginning of mosquito locomotor activity studies, investigations were carried out using an acoustic flight recording equipment created by Jones (1964) and adapted in the following years (Jones et al. 1967; Charlwood and Jones 1979; Peterson 1980; Rowley et al. 1987). This approach was followed by a commonly used system known as locomotor activity monitor (LAM–TrikNetics) model, which functions on break-beam detection through infrared light sensors (Eaton 1980).

In more recent studies, real-time detection and recording of insect movement have been explored using high-resolution cameras (Fry et al. 2000; Araujo et al. 2020; Ziegler et al. 2022). Guo et al. (2017) developed a device called FlyBox, which was designed to study the locomotor activity of *Drosophila*. Subsequently, Araujo et al. (2020) applied FlyBox to monitor the locomotor activity of male and female mosquitoes, specifically the vector species *Anopheles gambiae* and *Aedes aegypti*. Additionally, Wolkoff et al. (2023) used FlyBox to verify the effects of light pollution on the activity rhythms of the mosquito vector *Culex pipiens*.

The advancement of technologies and equipment capable of automatically tracking locomotor patterns of insects has allowed researchers to study different aspects related to this behavior in laboratory settings (Spitzen and Takken 2018). As a result, it has been confirmed that the locomotor activity of several mosquito species can be affected by physiological factors, such as their insemination state (Jones and Gubbins 1978; Jones 1981; Rowland 1989; Gentile et al. 2013; Lima-Camara et al. 2014; Araujo et al. 2020; Traoré et al. 2021), level of nutrition (Gentile et al. 2013; Lima-Camara et al. 2014; Araujo et al. 2020; Traoré et al. 2021) or infection state (Lima-Camara et al. 2011; Tallon et al. 2020; Padilha et al. 2018). However, no studies to date have been conducted on locomotor activity of the major malaria vector in the Amazon region, *Anopheles darlingi*.

*Anopheles darlingi* is the predominant malaria vector in most areas of the Amazon region (Tadei and Thatcher 2000; Gil et al. 2003) and exhibits nocturnal habits, with crepuscular activity peaks, highly anthropophilic behavior and susceptibility to human malaria parasites (Consoli and Oliveira 1994; Gil et al. 2015; Carlos et al. 2019). The available biological information about periodicity, time of activity and feeding behavior of *An. darlingi* was obtained through field works (Gil et al. 2007, 2015; Gama et al. 2009; Andrade et al. 2021).

The establishment of an *An. darlingi* colony in the Western Brazilian Amazon region in 2018 (Araujo et al. 2019) allowed us to develop studies under controlled environment, including investigations of the locomotor activity of this mosquito vector. Studying whether the behavior of *An. darlingi* is modulated by different physiological changes remains an important step towards the understanding of its biology and the development and/or evaluation of vector control tools in the field.

The importance of the vector *An. darlingi* in malaria transmission and the gap of biological information about its behavior has led us to study the effects of the main physiological aspects of anopheline life, blood feeding, insemination, and *Plasmodium* infection. In the present work, we evaluated the pattern of locomotor activity of the malaria vector *An. darlingi* under different physiological states at laboratory conditions. The results showed here will serve as a basis for future investigations into the molecular aspects involved in the endogenous regulation of the circadian rhythm of the *An. darlingi s*pecies.

## Methods

### Mosquito rearing

All experiments were carried out with female mosquitoes from a colony of *An. darlingi* maintained in PIVEM/FIOCRUZ-RO (Araujo et al. 2019). Mosquitoes were reared from larvae to adults under 12 h of light and 12 h of darkness (LD), at a temperature of 26 ± 1 °C and relative humidity of 70% ± 10%. Larvae were fed daily with TetraMin® Marine fish food and adults were fed with 15% honey solution ad libitum. Three- to 10-day-old females were used in the experiments.

### Insemination and blood-feeding experiment

To assess the effect of insemination on the locomotor activity of *An. darlingi*, virgin, and inseminated female mosquitoes were used. To assure that newly emerged females would not mate (virgin group), the cage with pupae was checked every hour to remove males. In order to obtain inseminated females, we allowed that emerged adult males and females mated freely in the cage for 6 days. The insemination was confirmed by dissection of female spermathecae and visual inspection under microscopy for the presence of sperm.

Experiments to assess the effect of blood feeding on the locomotor activity of females were initiated 30 min after blood feeding, which was performed using an artificial feeding membrane (Hemotek®) after the previous fasting period (≥ 12 h).

Therefore, there were four distinct groups of *An. darlingi* females regarding locomotor activity analysis: unfed virgin, blood-fed virgin, unfed inseminated, and blood fed inseminated. Experiments in Flybox were performed with 12 individuals per group and were repeated five times.

### Artificial infection experiment

Blood samples from malaria vivax patients were prepared for membrane-feeding assays. Each blood sample, collected in heparinizes vacutainer, was centrifuged at 1500 rpm for 10 min, and the plasma was replaced for an equal volume of inactivated human AB^+^ serum to reduce potential effects of human immunity and hence maximize the number of successfully infected mosquitoes. An aliquot of this blood sample was incubated at 42 °C for 30 min (Sangare et al. 2013).

Cages containing 60 female mosquitoes being 3 to 5 days old were placed under Hemotek feeders to allow blood feeding for 30 min. After that period, only fully fed mosquitoes were kept in the experimental cages. 15% honey solution on cotton wool pads were offered daily to the mosquitoes until midguts were dissected on the 7th day post-blood meal (pbm), then on the 10th day pbm locomotor activity assays started. After this procedure, there were two distinct groups of *An. darlingi* females to assess the *P. vivax* effect on locomotor activity: the mosquitoes fed with infected blood (*P. vivax*^+^) and those fed with uninfected blood (*P. vivax*^−^).

### Successful experimental infection and heat inactivation

At day 7 pbm, ten mosquitoes were dissected to confirm that infection and heat inactivation were successful. Midguts were dissected in PBS 1X, stained with 0.2% commercial mercurochrome (SIGMA) and examined for the presence of oocysts using microscopy (× 10). The rest of the mosquitoes remained in the original cage until the locomotor activity assays, which started at 10-day pbm and concluded in the 14-day pbm. After locomotor activity assays, salivary glands of all mosquitoes were dissected in RPMI to confirm sporozoite infection.

### Locomotor activity assays

In the 24-well plate, a piece of cotton soaked in 15% honey solution was placed at the bottom of each well, and individual anesthetized mosquitoes were added to each well. Then the plate was covered with a transparent plastic film, with holes poked over each well to promote air circulation. FlyBox, which is an automated video recording system developed to detect activity and sleep patterns in adult *Drosophila* flies (Guo et al. 2017), was used to monitor the locomotor activity profile of *An*. *darlingi*. This system has also been used to assess the locomotor activity patterns of two important tropical disease vectors, *An. gambiae* and *Ae. aegypti* (Araujo et al. 2020)*.*

The locomotor activity was monitored for up to 4 days using the Flybox system under a 12-h light/12-h dark photoperiod condition. Images of 24-well plate within the FlyBox were captured every 10 s and saved using the WebCan Image Save software (1.11). These images were then converted into video and the distance traveled by each mosquito was analyzed using PySoLo (version 1.1) (Gilestro and Cirelli 2009). The generated data were converted into a.txt file for analysis in the MatLab® software (version R2015b) (Donelson et al. 2012), which included locomotor activity quantification, rhythmicity, and periodicity, as described by (Araujo et al. 2020).

### Data analysis

The study analyzed the average locomotor activity of mosquitoes over a period of 3 consecutive days. The first 12 h of activity under LD conditions were excluded from the analysis to mitigate effects of the anesthesia from the ice-cooling. The mean locomotor activity was calculated for the following time intervals: the completed 24-h cycle (ZT0 to ZT23), photophase (ZT0 to ZT11), scotophase (ZT12 to ZT23), principal peak of activity (ZT12), and secondary peak (ZT0). The normality of data was assessed using the Shapiro–Wilk test. The analyses comparing time intervals were performed using the T-Student and Mann–Whitney tests. Graphs were generated in Excel (2016) and the statistical analysis was performed using GraphPad Prism software version 9.1.

## Results

**Fig. 1:**
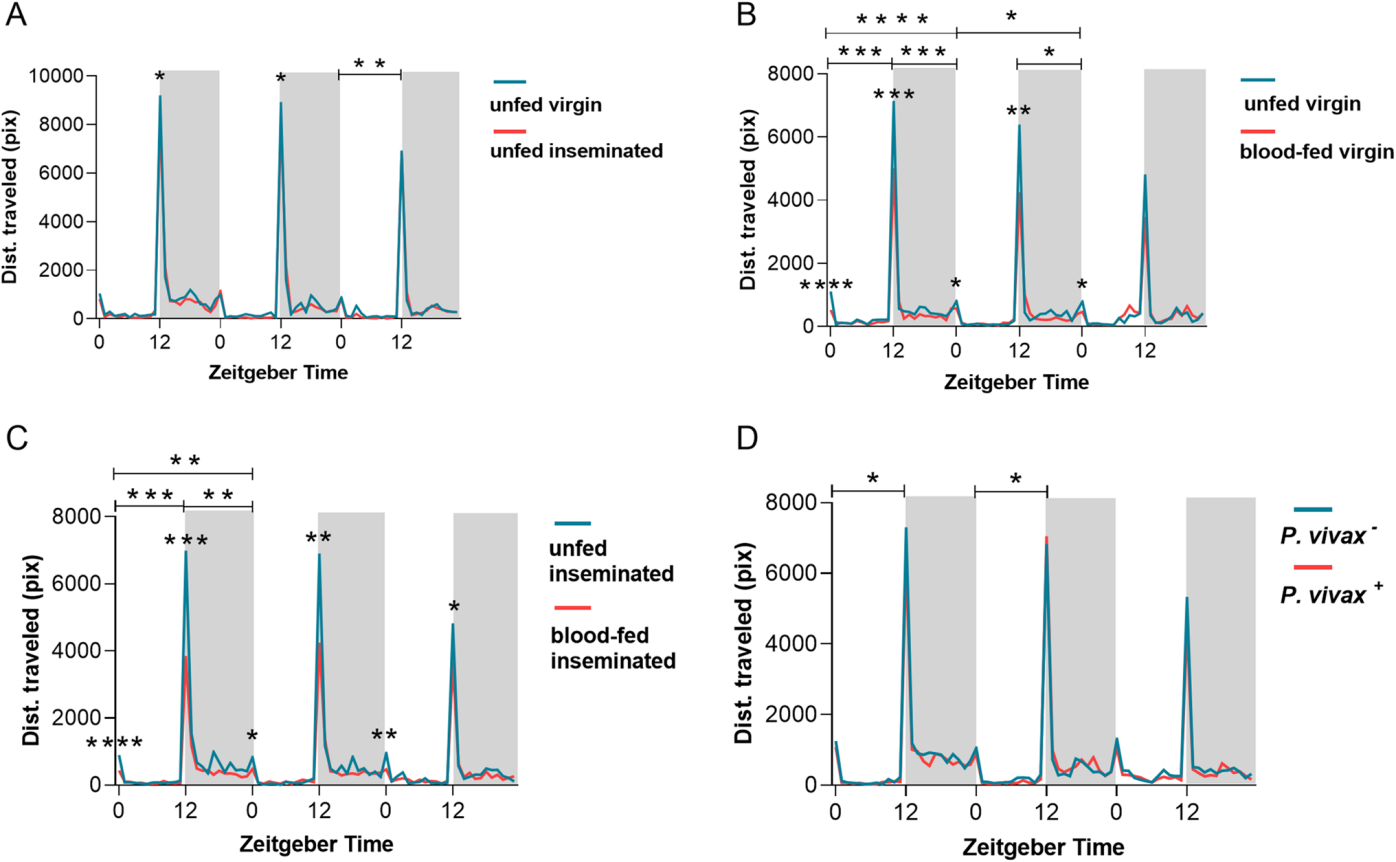
Average profile of the locomotor activity of *Anopheles darlingi* females during 3 days of analysis for each physiological condition: **A** unfed virgin vs. unfed inseminated; **B** unfed virgin vs. blood-fed virgin; **C** unfed inseminated vs. blood-fed inseminated; **D** infected *P. vivax*^+^ vs. uninfected *P. vivax*.^−^. White column represents the photophase and gray column, the scothophase. Time is measured in Zeitgeber (or ZT) hour, ZT0 being the onset of lights on and ZT12 being the onset of lights off as a 12-h light, 12-h dark schedule inside the Flybox. The data correspond to five independent experiments. Asterisks indicate statistical significance: *p < 0.05; **p < 0.01; ***p < 0.001; ****p < 0.0001

The graphs in Fig. 1 show the average locomotor activity profile of *An. darlingi* females under LD conditions for 3 consecutive days. In general, these mosquitoes exhibited nocturnal behavior with two distinct peaks, one during the scotophase (ZT12) and a second less intense peak at the beginning of photophase (ZT0).

The analysis of the locomotor activity of females under different insemination conditions revealed that unfed virgin females exhibit greater activity than unfed inseminated ones (Fig. 1A), with statistical differences in the ZT12 of the first and second day, as well as during the photophase on the third day (see supplementary Table S1). Spermatheca dissection showed that 100% of females from the inseminated group were positive to sperm presence and 100% of virgin females were negative.

When virgin females were subjected to blood feeding and compared to unfed virgin ones, it could be noticed that the unfed virgin females had significantly higher activity levels in all time intervals analyzed on the first and second day, except by the photophase on the second day (Fig. 1B). On the third day, the higher activity levels of unfed virgin females were only significant at ZT0, although this group still had higher locomotor activity when compared to the blood-fed virgin group (Fig. 1B). A similar pattern was observed in the analysis of locomotor activity of inseminated females which were either blood-fed or blood-unfed (Fig. 1C). Unfed inseminated females exhibited greater locomotor activity in all time intervals of the first day and higher activity levels was observed at ZT0 and ZT12 on subsequent days (Fig. 1C; see supplementary Table S1).

Regarding the locomotor activity of mosquitoes infected with *P. vivax* and uninfected ones, a statistical difference was registered in the photophase of the first and second day of analysis, the uninfected females showing greater locomotor activity (Fig. 1D; see supplementary Table S1).

**Fig. 2:**
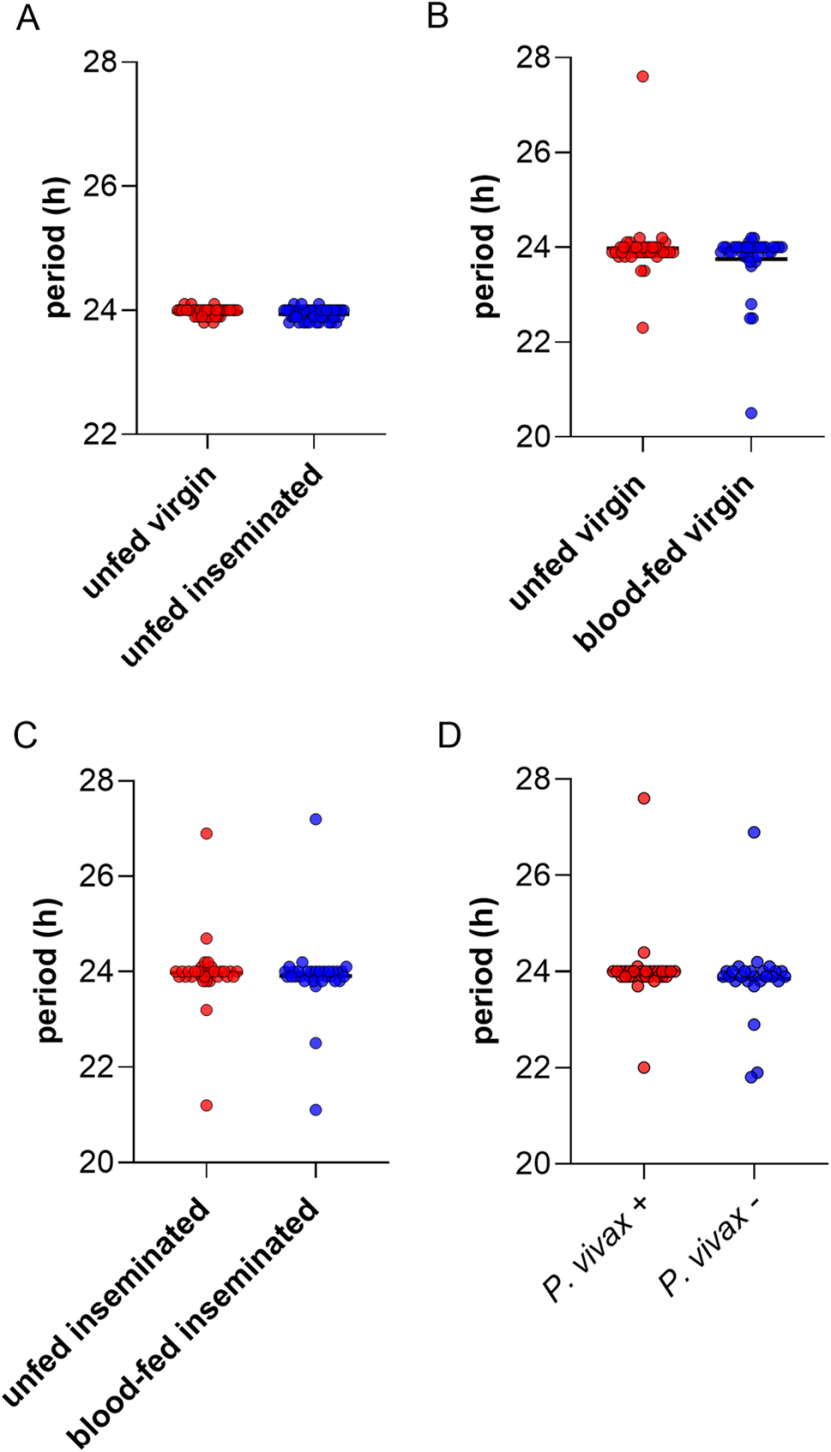
Average duration of the period in hours of locomotor activity of *Anopheles darlingi* females for each physiological condition: **A** unfed virgin vs. unfed inseminated; **B** unfed virgin vs. blood-fed virgin; **C** unfed inseminated vs. blood-fed inseminated; **D** infected *P. vivax*^+^ vs. uninfected *P. vivax*^−^. The data correspond to five independent experiments

**Fig. 3:**
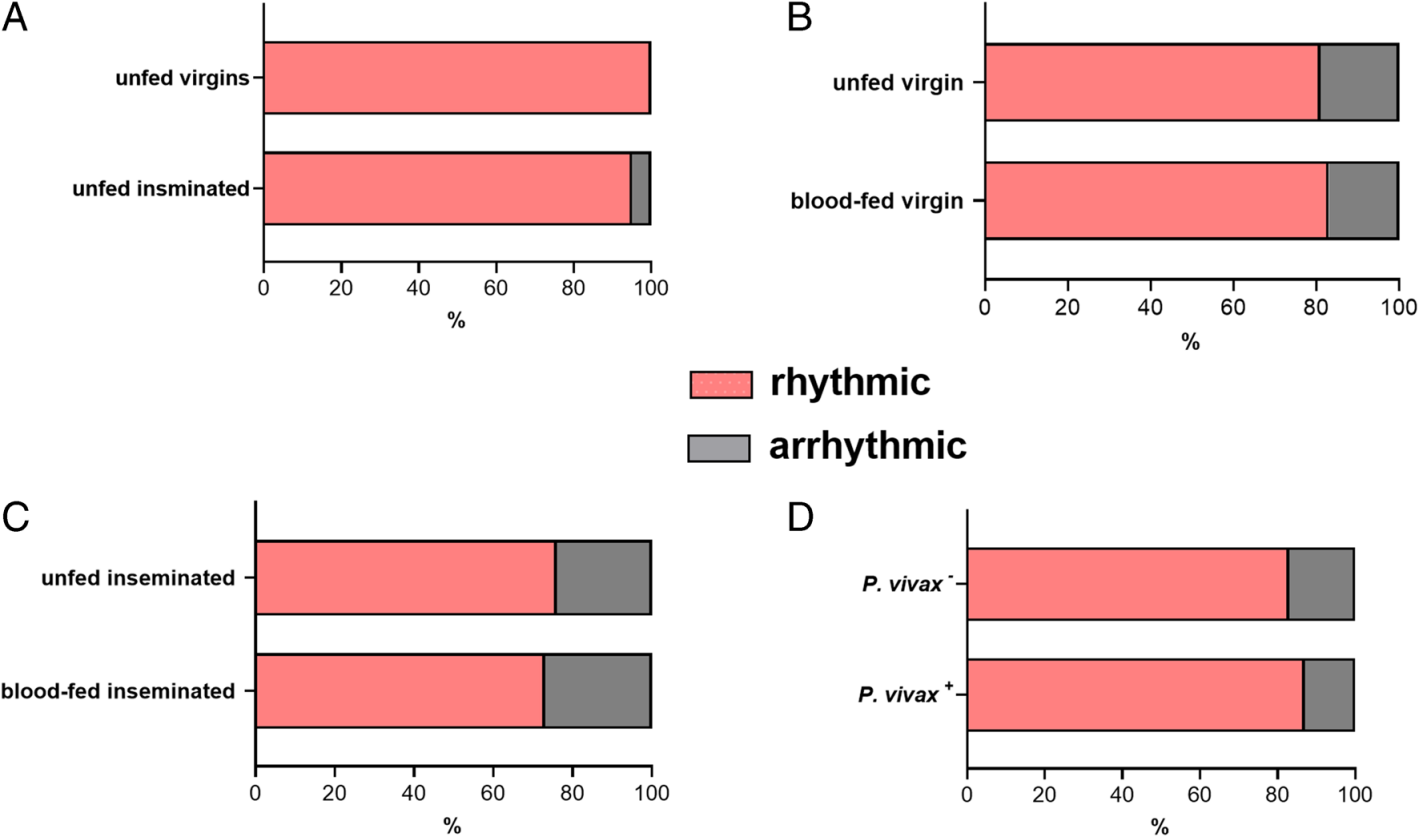
Quantification (%) of rhythmic (pink) and arrhythmic (gray) *Anopheles darlingi* females for each physiological condition: **A** unfed virgin vs. unfed inseminated; **B** unfed virgin vs. blood-fed virgin; **C** unfed inseminated vs. blood-fed inseminated; **D** infected *P. vivax*^+^ vs. uninfected *P. vivax*^−^. No statistically significant differences were observed among all groups. The data correspond to five independent experiments

Comparison of circadian periodicity was also performed under physiological conditions. As expected, all experimental groups exhibited an average circadian periodicity of approximately 24 h, and there was no significant difference observed between groups (Fig. 2). The rhythmicity of female mosquito groups (virgin and inseminated) was above 92% (Fig. 3A). For the groups designed to assess the impact of blood meal on the locomotor activity, the rhythmicity was above 81% for both virgin and inseminated females (Fig. 3B, C). The *P. vivax*^+^ mosquito groups exhibited rhythmicity above 73% (Fig. 3D). No statistical difference was observed in the rhythmicity comparisons between the groups tested.

Supplementary Table S2 presents the total number of mosquitoes analyzed for locomotor activity, periodicity, and rhythmic and arrhythmic cycles for each experimental group. Mortality was less than 35% for all physiological conditions in each experimental group (see supplementary Table S2).

## Discussion

This is the first report that evaluated the locomotor activity, rhythm, and the influence of different physiological states of *An. darlingi*, the main malaria vector in the Amazon region, under laboratory conditions. Similar studies have been described for other anopheline species (Jones and Gubbins 1978; Rowland 1989; Araujo et al. 2020; Traoré et al. 2021), as well as other mosquito vectors, including those from the genus *Culex* (Jones and Gubbins 1979; Chiba et al. 1990, 1992; Newman et al. 2016) and *Aedes* (Lima-Camara et al. 2011, 2013, 2014; Feitoza et al. 2020). The reason why there had been no circadian research on *An. darlingi* species is that colonization of this species had only been achieved in the last decade (Moreno et al. 2014; Villarreal-Treviño et al. 2015; Araujo et al. 2019; Puchot et al. 2022). Thus, the availability of *An. darlingi* colonies will improve the development of studies and provide further knowledge about the circadian rhythm and the biology of this vector.

In this study, *An. darlingi* exhibited a bimodal and predominantly nocturnal activity profile under LD conditions. The duration of activity period was found to be around 24 h, which was consistent with other anopheline species under 12-h light and dark cycles (LD 12-h photoperiod) (Jones et al. 1967, 1974; Sampaio et al. 2017; Duffield et al. 2019; Araujo et al. 2020; Traoré et al. 2021;). This bimodal pattern and nocturnal activity are recorded in field studies; however, the peaks of activity could vary depending on local geographical characteristics, climate, and human presence (Charlwood and Jones 1979; Forattini 1987; Tadei et al. 1998; Gil et al. 2007; Gama et al. 2009; Andrade et al. 2021).

*Anopheles darlingi* is considered eurigamous, like other anopheline species (Charlwood and Jones 1979; Consoli and Oliveira 1994; Diabate et al. 2003; Rund et al. 2012) whose females become refractory to further insemination after copulation (Baimai and Green 1987; Forattini 2002; Baldini et al. 2012; Baeshen 2022). The intense activity peak of virgin females observed at ZT12 in the present study coincides with the formation of mating swarms initiated by males in the dusk (Forattini 2002; Rund et al. 2012; Consoli e Oliveira 1994; Jones et al. 1967; Charlwood and Jones 1979). Such overlap in the locomotor activity of males and females of *An. darlingi* was observed in the experiment comparing both sexes under virgin condition (supplementary Figure S1). For inseminated females, this same peak at ZT12 may indicate an intense search for blood meal to proceed with the next stages of the gonotrophic cycle (Clements 1992; Baldini et al. 2012).

The reduction in locomotion of female mosquitoes after insemination corroborates previous findings for *An. gambiae* (Jones et al. 1967; Jones and Gubbins 1977, 1978; Araujo et al. 2020), *An. stephensi* (Rowland 1989), *Ae. aegypti* (Lima-Camara et al. 2014; Jones 1981), and mosquito vectors from the genus *Culex* (Jones and Gubbins 1979; Chiba et al. 1990, 1992). These mosquitoes showed several behavioral and physiological changes after mating due to the substances of the male accessory glands that had been transferred to females during copulation (Klowden 1999). The modulation of locomotor activity was already observed in virgin females that received a small dose of accessory gland solution by intrathoracic injection (Lima-Camara et al. 2013).

Overall, regarding physiological state (inseminated or virgin), the mosquitoes showed a significant decrease in locomotor activity after feeding on blood. This reduction in locomotor activity after blood meal is thought to be related to biological factors of blood digestion in mosquitoes and is consistent with previous studies (Jones and Gubbins 1978; Rowland 1989; Meireles-Filho et al. 2006). The timing of the reduction in locomotor activity (on the first and second days after feeding) suggests that the first 48 h of blood digestion may be more critic to *An. darlingi* (Consoli and Oliveira 1994; Forattini 2002). Inseminated females showed a similar decrease in locomotor activity after blood feeding, which is likely due to the energetic requirements of the oogenesis process, thus the mosquito would remain less active until oviposition, which generally occurs 72 h after blood meal (Jones and Gubbins 1978; Rowland 1989; Consoli and Oliveira 1994; Forattini 2002). The reduction on locomotor activity is related to a sensitivity reduction to human odors. Blood feeding can inhibit host search in female mosquitoes of the *An. gambiae* species (Takken et al. 2001) and is associated to genic regulation of chemosensory genes in *Ae. aegypti* (Hill et al. 2019) and *Culex quinquefasciatus* (Taparia et al. 2017).

Another important aspect to mention is that blood feeding is a behavior regulated by the circadian clock (Das and Dimopoulos 2008). Molecular evidence has shown changes in the expression of key circadian clock genes (*per*, *tim*, *cyc*, *clk*) in engorged vector insects that exhibit reduced locomotion (Meireles-Filho et al. 2006; Gentile et al. 2013). The authors suggest that this may be a response to oxidative stress during blood digestion. With respect to the *An. gambiae* vector, it has been described that some molecular regulations related to detoxification processes of products generated during blood digestion are rhythmic (Rund et al. 2011, 2016). However, it is not yet clear by which mechanisms oxidative stress affects endogenous circadian control.

The primary behavioral changes in anophelines resulting from infection occur during the infective stages of *Plasmodium* sporozoites (Rowland and Boersma 1988; Anderson et al. 1999; Cator et al. 2013; Thiévent et al. 2019). Many of these changes, such as increased blood meal frequency (Wekesa et al. 1992; Koella et al. 1998; Koella and Packer 1996; Ferguson and Read 2004) and heightened response to host odors (Smallegange et al. 2013; Cator et al. 2013, 2015), are closely tied to the locomotion of the anophelines. Experiments with the *Ae. aegypti* vector have revealed not only changes in feeding behavior (Maciel-De-Freitas et al. 2013; Sylvestre et al. 2013) but also modifications to their locomotor activity profile. Specifically, female mosquitoes infected with dengue virus serotypes 1 and 2 become more active (Lima-Camara et al. 2011; Tallon et al. 2020), whereas those infected with Zika virus show a reduction in the activity of females of this species (Padilha et al. 2018).

From the results obtained in this study, we do not consider that the statistically significant difference recorded indicates biologically relevant changes in the locomotor activity of *An. darlingi* females in view of *P. vivax* infection*.* This is mainly because *An. darlingi* is a nocturnal mosquito species, and the data presented here showed a natural reduction in activity during a diurnal period, when it is typically less active (Gama et al. 2009; Gil et al. 2007; Andrade et al. 2021; Villarreal-Treviño et al. 2015). Among the studies about the effects of *Plasmodium* infection on mosquito locomotor activity, only one recorded a small reduction in locomotion of *An. stephensi* mosquitoes infected with the rodent malaria parasite *P. yoelii*, starting the monitoring on day 9 post-infection (Rowland and Boersma 1988). Competition for nutrients and tissue damage during parasite development are possible causes for the reduction in locomotion. In another study (Vantaux et al. 2015) which used long and short-range techniques (olfactometry and recording of locomotor activity), no significant differences were found in the group of *An. coluzzi* females infected by *P. falciparum* compared to the uninfected control group. Studies evaluating *Plasmodium* infection on the locomotor activity of anopheline mosquitoes are scarce, which limits our understanding of how the parasite affects this rhythm in the malaria vector. Therefore, in the future, it will be of interest to assess the locomotor activity of *An. darlingi* at different stages of *P. vivax* development to better understand the effects of the parasite on this important behavior in the malaria vector.

## Conclusions

In conclusion, *An. darlingi* exhibits bimodal, mainly nocturnal locomotor activity. Insemination and blood feeding are among the physiological factors that influence this malaria vector’s locomotor activity. However, *P. vivax* infection does not significantly alter the locomotor activity of *An. darlingi* in a manner that may affect the disease transmission under experimental conditions. These data will be essential for further studies of locomotor activity and circadian rhythms of this vector, particularly at the molecular level.

### Supplementary Information

Below is the link to the electronic supplementary material.

Supplementary file1 (DOCX 22 KB)

Supplementary file2 (DOCX 19 KB)

Supplementary file3 (TIF 244 KB)

## Data Availability

All data generated or analyzed during this study are included in this published article and its supplementary information files.

## Abbreviations

*PIVEM*: Malaria Vector Production and Infection Platform
*LD*: Light and dark
*LAM*: Locomotor activity monitor
*pbm*: Post-blood meal
