# Supplementary material for: Evaluation of insemination, blood feeding, and Plasmodium vivax infection effects on locomotor activity patterns of the malaria vector Anopheles darlingi (Diptera: Culicidae)
Source: Parasitol Res. 2023 Dec 7;123(1):15. doi: 10.1007/s00436-023-08053-5 (PMC10703739; doi:10.1007/s00436-023-08053-5)
Supplement: Supplementary file 1 — Supplementary file1 (DOCX 22 KB) [file 436_2023_8053_MOESM1_ESM.docx]

Table S1. Statistical difference in the various time intervals showed in Figure 1.

| Physiological condition | Day | Time intervals | Statistical analysis |
| --- | --- | --- | --- |
| Unfed Virgin vs. Unfed Inseminated | First | ZT12 | t= 2.442, df= 86, P= 0, 0167 |
|  | Second | ZT12 | t=2, df= 86, P= 0.0309 |
|  | Third | Photophase | U= 624, P= 0.0091 |
|  | First | 24 cycle | t= 4.325, df= 92, P< 0.0001 |
|  | Second | 24 cycle | U= 846, P= 0.0254 |
|  | First | Photophase | U= 627, P= 0.0002 |
|  | First | Scotophase | t=3.562, df= 95, P= 0.0006 |
| Unfed virgin vs. Blood-fed virgin | Second | Scotophase | U= 973, P= 0.1958 |
|  | First | ZT12 | t= 3.800, df= 94, P= 0.0003 |
|  | Second | ZT12 | U= 724, P= 0.0016 |
|  | First | ZT0 | U= 552.5, P< 0.0001 |
|  | Second | ZT0 | U= 820.5, P= 0.0151 |
|  | Third | ZT0 | U= 839, P= 0.0221 |
|  | First | 24 cycle | U= 549.5, P= 0.0011 |
|  | First | Photophase | U= 546, P= 0.0010 |
|  | First | Scotophase | U= 574, P= 0.0023 |
| Unfed inseminated vs. Blood-fed inseminated | First | ZT12 | U= 501.5, P= 0.0002 |
|  | Second | ZT12 | U= 569, P= 0.0020 |
|  | Third | ZT12 | U= 650.5, P= 0.0183 |
|  | First | ZT0 | U= 455, P< 0.0001 |
|  | Second | ZT0 | U= 685.5, P= 0.0402 |
|  | Third | ZT0 | U= 615.5, P= 0.0075 |
| Infected with *P. Vivax* vs. Uninfected | First | Photophase | U= 687.5, P= 0.0312 |
|  | Second | Photophase | U=668.5, P= 0.0203 |
