# Supplementary material for: Evaluation of insemination, blood feeding, and Plasmodium vivax infection effects on locomotor activity patterns of the malaria vector Anopheles darlingi (Diptera: Culicidae)
Source: Parasitol Res. 2023 Dec 7;123(1):15. doi: 10.1007/s00436-023-08053-5 (PMC10703739; doi:10.1007/s00436-023-08053-5)
Supplement: Supplementary file 2 — Supplementary file2 (DOCX 19 KB) [file 436_2023_8053_MOESM2_ESM.docx]

Table S2. Total number of individuals in each experimental group.

| Physiological condition | Meal | N starting | N dead  (%) | Total N analyzed | N rhythmic (%) | N arrhythmic (%) |
| --- | --- | --- | --- | --- | --- | --- |
| Virgin | Glucose | 60 | 18 (30.0) | 42 | 39 (92.9) | 03 (7.1) |
| Inseminated | Glucose | 60 | 14 (23.3) | 46 | 46 (100) | 0 (0) |
| Virgin | Glucose | 60 | 12 (20.0) | 48 | 39 (81.3) | 09 (18.8) |
| Virgin | Blood | 60 | 14 (23.3) | 46 | 38 (83.0) | 08 (17.4) |
| Inseminated | Glucose | 60 | 19 (31.7) | 41 | 31 (75.6) | 10 (24.4) |
| Inseminated | Blood | 60 | 15 (25.0) | 45 | 33 (73.3) | 12 (26.7) |
| Uninfected | Uninfected blood | 60 | 13 (21.7) | 47 | 41 (87.2) | 6 (12.8) |
| Infected | Infected blood | 60 | 20 (33.3) | 40 | 33 (82.5) | 7 (17.5) |
