# Supplementary figures and images for: Evaluation of insemination, blood feeding, and Plasmodium vivax infection effects on locomotor activity patterns of the malaria vector Anopheles darlingi (Diptera: Culicidae)
Source: Parasitol Res. 2023 Dec 7;123(1):15. doi: 10.1007/s00436-023-08053-5 (PMC10703739; doi:10.1007/s00436-023-08053-5)

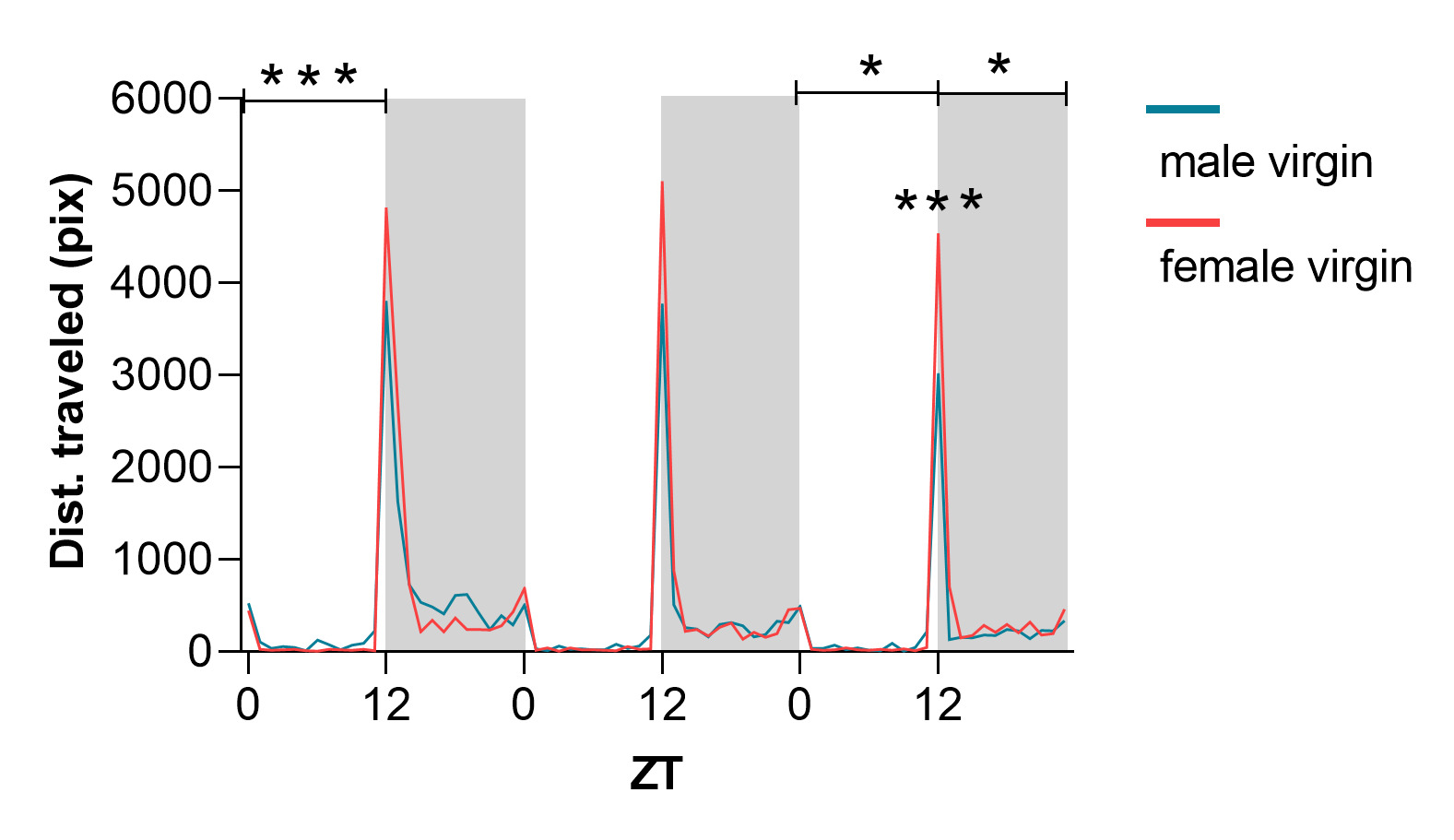

Supplement: Supplementary file 3 — Supplementary file3 (TIF 244 KB) [file 436_2023_8053_MOESM3_ESM.tif]
